# Whom to trust? Inferred source credibility and response borrowing in a memory task

**DOI:** 10.3758/s13421-023-01423-5

**Published:** 2023-04-21

**Authors:** Aleksandra Krogulska, Kinga Izdebska, Maciej Hanczakowski, Katarzyna Zawadzka

**Affiliations:** 1 Institute of Psychology, Jagiellonian University, Kraków, Poland; 2 Department of Psychology, University of Warwick, Coventry, UK; 3 Faculty of Psychology and Cognitive Science, Adam Mickiewicz University, Poznań, Poland; 4 Interdisciplinary Center for Applied Cognitive Studies, SWPS University, Warszawa, Poland

**Keywords:** Credibility, Response borrowing, Conformity

## Abstract

We investigated whether people can discriminate between sources of information that are either credible or respond at random, based only on their own knowledge and the responses provided by these sources. In three experiments, participants were asked to judge the validity of trivia statements. Some statements were accompanied by true/false responses provided by either a credible source or a source whose responses were random. In Experiment 1, participants first saw a set of easy questions, which provided the basis for assessing the relative credibility of the sources, before responding to a set of difficult questions, where response borrowing was assessed. In Experiments 2 and 3, participants solved a test composed of difficult questions only, but only after studying the correct responses to all these questions. In Experiment 2, there was no delay between the study and test phases, whereas in Experiment 3, the delay was 24 hours. In all experiments, more participants explicitly identified the more credible source in the postexperimental questionnaire than misidentified the noninformative source as credible. However, differentiated response borrowing—borrowing more responses from the credible than the noninformative source—emerged only in Experiment 2. Therefore, people can often explicitly infer source credibility from the responses the sources provide. However, using these inferences to regulate response borrowing is relatively less likely and happens only under specific, favorable circumstances.

Nowadays, any person connected to the internet can access a massive amount of information, and human communication has never been so fast. However, we also face a vast amount of misinformation and fake news, which not only lead people astray but also undermine their beliefs in facts (e.g., Lewandowsky et al., 2017). For instance, despite extensive scientific evidence for the climate crisis or the high efficiency of vaccines, many people deny these facts and continuously seek confirmation of their attitudes based on unverified sources of information (see, e.g., Kunda, 1990). Moreover, new ways of human communication enable almost anyone to spread information without any restrictions. Thus, it is of utmost importance to establish whether and, if so, to what extent people can discriminate between sources of high and dubious credibility. Here, we look at the extent to which people are able to (1) verify credibility of sources providing them with information and (2) attune the degree to which they use this information themselves when facing a memory task.

Research on source credibility often uses one of two approaches. First, this research can manipulate source credibility directly, presenting labels describing sources as, for example, experts in a particular domain (Horry et al., 2012; Smith & Ellsworth, 1987), or as persons who have privileged access to some information of interest (French et al., 2011; Gabbert et al., 2007). The effect of such a direct manipulation on either explicit judgments of source credibility or processing of information conveyed by this source is then examined. This line of research looks at credibility as a peripheral cue, divorced from the message a particular source delivers, which plays its role particularly if the message itself is not properly processed (Petty & Cacioppo, 1986). A host of labeling manipulations, ranging from indicators of expertise (e.g., Van Boekel et al., 2017) to information concerning alcohol intake (Zajac et al., 2016), has been found to affect not only explicit judgments of credibility but also a variety of indirect measures, such as judgments of plausibility of a conveyed message (Wertgen & Richter, 2020), the extent to which external information is used as one’s own response (e.g., Andrews & Rapp, 2014), the time it takes to process this information, or subsequent information that is either consistent or inconsistent with the information provided by sources of varying credibility (Sparks & Rapp, 2011). All these studies confirm that credibility of a source robustly affects performance in a variety of tasks in which external information is available.

While it is often important to establish how a direct manipulation of credibility via labeling affects information processing, this approach suffers from one important shortcoming. In practice, any assertion concerning source credibility—such as information that someone is an expert—can often be as misleading as the message itself. This is particularly obvious nowadays given the preponderance of self-proclaimed experts providing false information—be it tech entrepreneurs discussing international politics or politicians promoting dubious health-care interventions. This is where the second line of research on credibility stems from. In this line of research, no labeling is used and credibility is instead manipulated by varying the properties of the message itself, such as its validity or plausibility. Here, the assumption is that credibility is not always peripheral to the message, and indeed the most straightforward way of inferring whether a source is credible is to assess the correctness of the message(s) this source conveys, based on some internal standard. For example, Collins et al. (2018; see also Madsen et al., 2020) found that when a source provides a plausible message, one that remains in agreement with participants’ knowledge about the world, this increases explicit judgments of credibility of this source, while an implausible message decreases the same judgments. Wertgen and Richter (2020) used the labeling manipulation, presenting their sources as experts or nonexperts, while also manipulating message plausibility. They also found that message plausibility affected explicit judgments of credibility, both when external sources were labeled as experts and nonexperts. These studies demonstrate that appraisal of the contents of a message and the source of this message is a bidirectional process. Not only does source credibility determine how messages are processed, but at the same time message processing allows for inferences regarding source credibility.

Studies varying source credibility by manipulating the content of the message can look at explicit assessments of credibility, but an arguably more important avenue of research concerns the impact of perceived source credibility on information processing. In this case, the focus is on the dynamics of the bidirectional relationship between content analysis and source credibility inferences. If a message is deemed implausible and thus reduces the credibility of the source that provided it, does it influence the processing of subsequent messages provided by the same source? Collins et al. (2018) indeed showed that plausibility of a message exerts further influence on processing subsequent messages provided by the same source. When a neutral statement followed either a plausible or an implausible statement provided by a source, this neutral statement was itself perceived as more or less plausible, respectively. This pattern suggests that the relationship between content analysis and inferred source credibility is not only bidirectional but also dynamic, one affecting the other in turns. However, in the particular task used by Collins et al., participants were asked to process only two statements. In many situations, people are exposed to a number of messages from a given source, with some of them being true and some false. The credibility of the source depends often on a particular mixture—the extent to which the source provides predominantly accurate information as opposed to guesses. Can people discern source credibility and inform their processing of messages this source conveys when they face a mixture of accurate and inaccurate statements?

At least two studies provided results that address the question posed here. Recent findings by Pescetelli and Yeung (2021) provide evidence for successful credibility inference and its further influence on information processing exactly under conditions in which multiple messages were provided by sources of varying credibility. In a perceptual decision task, participants not only adjusted their explicit assessments of source credibility in line with the actual credibility of the sources but also moderated the extent to which external advice influenced their own responding in the main task. Participants borrowed responses—and presented them as their own—more often from sources that, in the long run, generally agreed with their own initial decisions.^1^

Using a similar design, but substituting perception for an episodic memory task, a study by Jaeger et al. (2012; see also Zawadzka et al., 2016, for a replication, and Numbers et al., 2014, for related findings) found much less evidence for effective use of information concerning credibility. Jaeger et al. used a recognition memory task, in which participants studied lists of words and subsequently were asked to distinguish between studied and nonstudied items in the presence of responses coming from one or two external sources. These sources differed in the overall accuracy of their responses, with one source providing generally accurate ‘advice’ (75% correct), while the other responding at random (50% correct). The study showed that even though participants were often able to explicitly name the more accurate source in a postexperimental questionnaire (i.e., assess credibility explicitly), they borrowed responses as often from the credible as from the random or noninformative source. Only when the accuracy of one of the sources was below the chance level, at 25% correct responses, were participants less likely to borrow responses from this source compared with the credible source.

There is clearly a great difference between perceptual and memory decisions that may well be responsible for the varying patterns described here. For perceptual decisions, as studied by Pescetelli and Yeung (2021), one’s knowledge depends on the information immediately available, almost at the same time at which advice is provided by an external source. In this situation, what one has to do is to simply keep in memory the tally of agreements and disagreements with a particular source to discern its credibility. By contrast, for episodic memory, as studied by Jaeger et al. (2012), one deals with information that is not readily available, and thus external advice needs to be contrasted with information retrieved from memory rather than derived from the immediate environment. This requirement to use memory as a reference point for assessing external information may create an additional cognitive burden, interfering with remembering the tally of agreements and disagreements, thus undermining the ability to infer source credibility and use such inferences to guide response borrowing. If this is correct, then the involvement of memory as an internal standard for judging the veracity of external information puts a strong limit on the effectiveness of using content analysis for credibility inference.

In the present study, we aimed to further assess the effectiveness of credibility inference in tasks that require memory retrieval to judge the accuracy of multiple responses provided by external sources. While Jaeger et al. (2012) only showed highly constrained effects of credibility on response borrowing in their episodic memory task, we reasoned that one problem with generalizing their results could be related to the particular task they used—simple recognition of single words. Arguably, these impoverished materials may not provide strong memories of the sort that would support effective credibility inference. Consequently, in the present investigation, we focused on a task requiring assessing the validity of trivia facts rather than single words. Trivia facts constitute richer materials that should be of higher interest to participants and should engender stronger, more interconnected memory representations. Moreover, even if participants have no prior knowledge regarding the veracity of trivia statements, they can potentially resort to their preexisting knowledge when trying to guess whether the statement is correct or not; for example, one’s general knowledge of Greek mythology could allow for venturing a guess regarding a particular mythological figure. This should in turn allow for contrasting and assessing accuracy of advice coming from external sources. We thus assessed whether with this change in materials, it would be possible to detect the effects of message accuracy on credibility assessments and response borrowing.

In three experiments, participants were asked to judge whether trivia statements were true or false while being shown responses coming from two sources—presented as ‘previous participants’—which differed in the accuracy of information they conveyed: one source provided useful information, while another responded randomly. We examined the effects of this manipulation of source informativeness on explicit assessments of source credibility and on patterns of response borrowing from both sources. We decided to contrast a credible source with a noninformative rather than a misleading one because this comparison seems to have more bearing on how credibility inferences are arrived at in everyday-life situations: where the challenge is often to distinguish between real experts and pseudoexperts with no actual knowledge rather than obvious liars. Thus, we focus here on what can be described as expertise of external sources, as opposed to their trustworthiness, which is the extent to which sources can be trusted not to lie. We return briefly to this issue in General Discussion.

In Experiment 1, participants first responded to a relatively short series of easy questions and then took a test on a longer series of difficult questions. We assessed whether participants would derive information concerning source credibility based on the initial run of easy questions, and then would build on this inference to attune their response borrowing when faced with difficult questions. To foreshadow, the results failed to reveal a modulating effect of source credibility on response borrowing, replicating the previous findings from studies that employed a word recognition task (Jaeger et al., 2012; Zawadzka et al., 2016). In Experiments 2 and 3, the trivia test consisted only of difficult questions but was preceded by a study phase for the information included in these questions. This was done to provide a reference point for judging the accuracy of all information presented by external sources throughout the memory task. In Experiment 2, the test immediately followed the study phase, whereas in Experiment 3 participants completed it the next day. Under these conditions, participants adjusted their response borrowing to the credibility of external sources, but only with immediate testing implemented in Experiment 2. This provides evidence that a dynamic and bidirectional relationship between content analysis and credibility inference is possible also in a memory task, but only as long as a highly accessible standard for judging the accuracy of external information is provided.

## Experiment 1

The purpose of the present experiment was to assess whether participants can derive information concerning source credibility from a set of easy trivia questions requiring providing true/false judgments. When answering these easy questions, participants were provided with cues—true/false responses—coming from two external sources, one being correct on 83% of questions and the other on 50% of questions (chance-level accuracy). The ability to derive and use information regarding source credibility was examined by assessing whether participants attuned their response borrowing according to the actual credibility of the sources in a following set of difficult trivia questions, as well as by collecting explicit judgments of source credibility after the main experimental task.

### Method

#### Participants

Seventy-two students (62 female) between the ages of 18 and 50 years (*M* = 25.75, *SD* = 8.19) of the SWPS University (Poland) participated in the study. Jaeger et al. (2012, Experiment 2), on whose procedure the current study is based, revealed reliable effects of source credibility when credible and misleading sources were compared with data from 34 participants. We more than doubled this sample size, expecting the effects of interest to be smaller with a noninformative rather than a misleading source. Participants were tested online and received course credit for their participation. All participants were treated in accordance with the Polish National Science Centre guidelines for research ethics.

#### Design

In Experiment 1, all manipulations were employed within participants. The main manipulation concerned the cues presented at test. These cues could either be provided by the credible source (the *cued-credible* condition), the noninformative source (the *cued-noninformative* condition), or absent (the *uncued* condition). In the cued conditions, the cues could either be valid (i.e., consistent with the correct answer), or invalid, with a particular mixture of valid and invalid cues (83% vs. 17% or 50% vs. 50%) depending on the type of source. The dependent measures included the accuracy of participants’ recognition responses, the index of bias to provide responses congruent with external cues, and the rate of explicit identifications of the credible source in the postexperimental questionnaire.

#### Materials

For the initial experimental task, a set of easy trivia questions (e.g., *Who wrote “Romeo and Juliet”? Shakespeare*) was prepared based on a pilot study with a student population (*N* = 121). Here, 174 trivia questions were presented to participants in a true/false format and a set of 48 questions for which performance was the highest was chosen. The average performance for those 48 questions was 87%.

For the main experimental task, difficult trivia questions were collated from various online sources: Wikipedia, webpages with trivia questions, high-school textbooks, maps, and so on. We made every effort to ensure that the questions encompassed a broad thematic scope (e.g., history, geography, literature, fine arts, biology, mathematics, physics, linguistics). Answers to these questions consisted of a single word or phrase (e.g., year, name, common noun) and were emotionally neutral. We conducted a pilot study (*N* = 21) in which questions were presented in an open-ended format. Based on participants’ responses, 288 questions were chosen for which at most one participant in the pilot study answered correctly. In another pilot study (*N* = 24), in which we trialled these difficult questions in a true/false format, the average accuracy was 59% (with the chance level being 50%). All selected questions were split into 96 groups of three questions. These groups consisted of questions about a similar subject and having the same answer format—for example, *In Greek mythology, what was the name of the goddess of the moon?* (Selene); *What was the name of the Minotaur’s mother?* (Pasiphae); *In Greek mythology, what was the name of the goddess of magic spells, darkness, and ghosts?* (Hecate). Questions were assigned to be presented with either their correct responses or incorrect responses, and this assignment was counterbalanced between participants. For incorrect responses, all questions from a related triplet had incorrect responses, with responses recombined across questions to ensure their plausibility.

#### Procedure

Participants completed two tests in which they were asked to judge trivia statements as either true or false. The first test consisted of 48 easy trivia questions. The second test consisted of 288 difficult trivia questions.

In both tests, questions appeared randomly one at a time at the top of the computer screen, along with the true/false response options underneath. Participants’ task was to decide whether the presented answer was correct (by choosing ‘true’) or incorrect (by choosing ‘false’). There was no time limit to provide the answers. Half of the questions were presented with their correct answers, the other half with incorrect ones.

For the easy test, each question was presented with a cue provided by one of two external sources. One source provided accurate responses for 20 out of 24 questions (83% accuracy), whereas the other source provided accurate responses for 12 out of 24 questions (50% accuracy).^2^ Participants were asked to pay attention to the ‘true’ or ‘false’ cues provided by the sources. The assignment of questions to sources, as well as to ‘true’ and ‘false’ cues was counterbalanced across participants. Cues were presented simultaneously with questions; thus, participants did not have to remember them and could borrow them immediately by providing a response consistent with the cue. Participants were told that sources’ responses were provided by two participants who took part in the same experiment earlier. Responses of one source were accompanied by a cartoon depiction of a woman and other by a depiction of a man. Participants were told that these depictions did not necessarily reflect the gender of ‘previous participants’ as their only purpose was to make distinguishing the sources easier. The assignment of gender to the credible versus noninformative source was counterbalanced across participants. Questions, answers (correct or incorrect), and external cues were presented first, and participants were only able to answer the question after a 1-second delay. This delay was introduced so that participants would pay attention to the cue before answering the question.

**Fig. 1:**
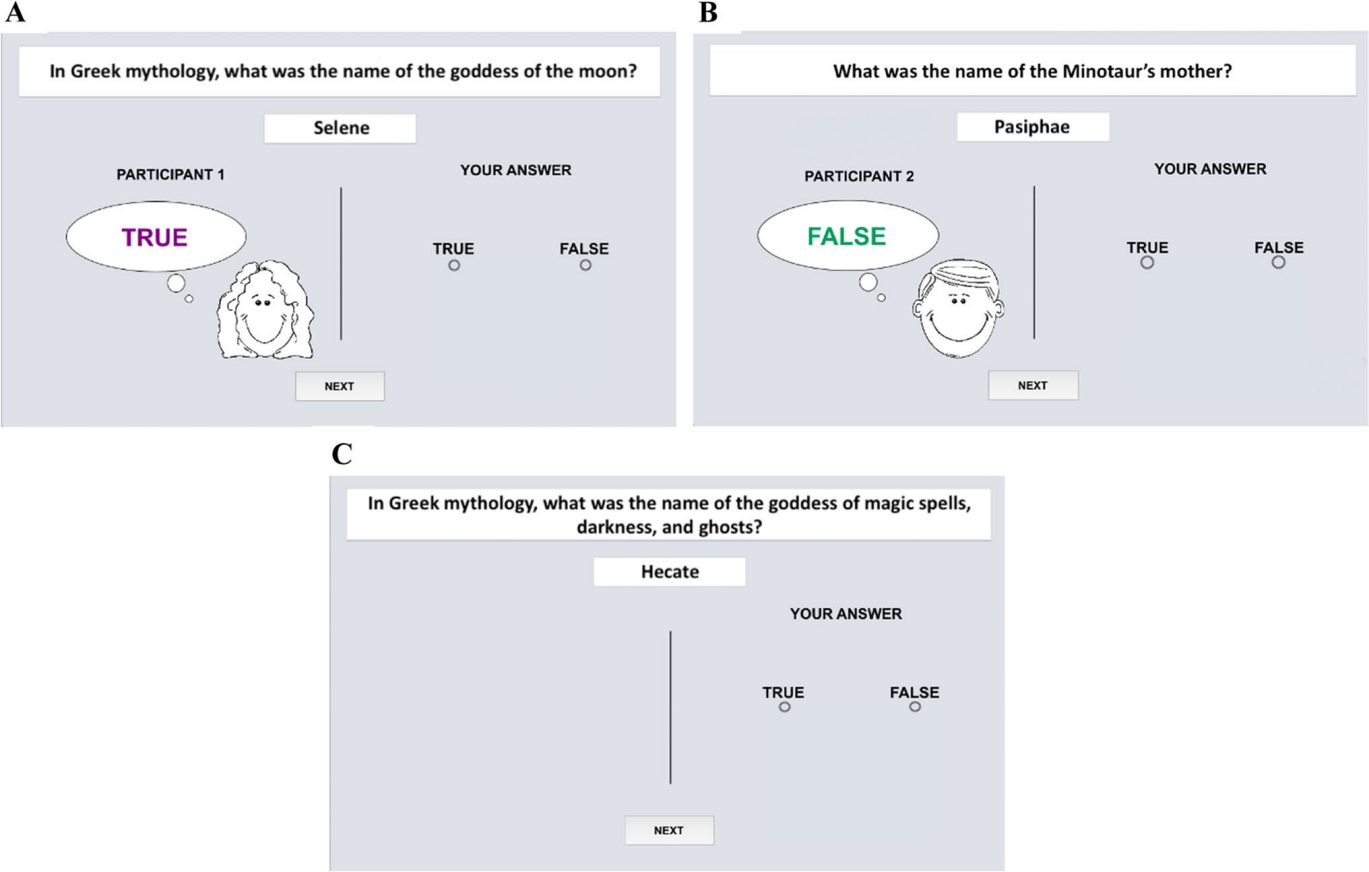
Examples of trial types across cuing conditions in Experiments 1–3 (translated into English). Panels **A** and **B** depict trials from the two cued conditions, with each of the cartoon faces representing the credible (vs. noninformative) source for half of the participants. Panel C depicts the uncued condition

The second test was identical to the first, except for the addition of questions for which no cues were presented (see Fig. 1 for an example of cued-credible, cued-noninformative, and uncued trials in the main test). Thus, the credible source provided correct cues for 80 out of 96 questions, the noninformative source provided correct cues for 48 out of 96 questions (chance-level accuracy), and the remaining 96 questions were uncued.

Upon completing the second test, participants were asked which of the sources gave more correct responses. The same cartoon pictures of a man and a woman that were used at tests were once again presented, and participants had to pick one of those sources as more credible.

### Results and discussion

#### Accuracy

As we expected, performance in the first test, with easy questions, was uniformly high. There was no difference in performance between the trials cued by the credible (*M =* 0.90, *SD =* 0.08) and noninformative (*M =* 0.89, *SD =* 0.09) source, *t*(71) = 0.62, *SE* = 0.01, *p* = .539, *d* = 0.08. This high performance suggests that participants—at least in principle—should have been able to use their own knowledge to determine the veracity of the sources’ cues.

In the second test, following Jaeger et al. (2012; see also Zawadzka et al., 2016), a signal-detection measure of *d′* (hit rate minus false-alarm rate, both transformed into standard deviation units − *z*-scores) was computed to assess test performance. The descriptive statistics for *d′* are presented in Table 1. First, *d′* scores were analyzed as a function of cuing condition (cued-credible, cued-noninformative, uncued) with a repeated-measures analysis of variance (ANOVA). This revealed significant differences across conditions, *F*(2, 142) = 44.97, *MSE* = 0.16, *p* < .001,${\eta}_{\textrm{p}}^2$ = .388. Follow-up *t* tests revealed that when cues were provided by the credible source, accuracy was improved—*d′* was higher—compared with the baseline uncued condition, *t*(71) = 6.89, *SE* = 0.08, *p* < .001, *d* = 1.00. However, accuracy when presented with cues from the noninformative source did not significantly differ from the baseline, *t*(71) = 1.00, *SE* = 0.05, *p* = .322, *d* = 0.12.

**Table 1:** Mean values of d′ across cuing conditions in Experiments 1–3

Experiment	Cued–Credible	Cued–Noninformative	Uncued
Experiment 1	0.77 (0.63)	0.20 (0.41)	0.25 (0.37)
Experiment 2	2.16 (0.84)	1.97 (0.86)	1.96 (0.88)
Experiment 3	2.01 (0.76)	1.66 (0.81)	1.73 (0.73)

Standard deviations are in parentheses

The improvement in *d′* when presented with cues from the credible source compared with the uncued condition could only be produced if participants relied on the cues provided by that source. Note, however, that the lack of difference between the noninformative source and the baseline condition in terms of *d′* says nothing about participants’ reliance (or lack thereof) on cues provided by the noninformative source. Such a result would be expected if participants ignored the source completely, as well as if they borrowed responses from the source only in those cases in which their own performance would be at chance—what Jaeger et al. (2012) termed low-confidence outsourcing (see also Koop et al., 2021)—because that would only substitute participants’ chance-level responses with source’s chance-level responses. However, response borrowing can also be clearly evident in performance measures if these are compared across valid and invalid cues, where it should lead to an increase in performance for valid as opposed to invalid cues.^3^ A 2 (cue validity: valid, invalid) × 2 (source: credible, noninformative) repeated-measures ANOVA was performed on *d′* scores (see Table 2 for descriptive statistics). The ANOVA yielded only a significant effect of cue validity, *F*(1, 71) = 66.90, *MSE* = 2.35, *p* < .001, ${\upeta}_{\textrm{p}}^2$= .485, as *d′* scores were higher after valid (*M* = 1.02, *SD* = 0.89) than after invalid (*M* = −0.45, *SD* = 0.86) cuing. The main effect of source, *F*(1, 71) = 1.45, *MSE* = 0.29, *p* = .232, ${\upeta}_{\textrm{p}}^2$= .020, as well as the interaction, *F*(1, 71) = 1.81, *MSE* = 1.18, *p* = .183, ${\upeta}_{\textrm{p}}^2$ = .025, were not significant, confirming that participants borrowed responses from both sources.

**Table 2:** Mean values of d′ across source conditions and cuing validity in Experiments 1–3

Experiment	Credible source		Noninformative source	
	Valid cues	Invalid cues	Valid cues	Invalid cues
Experiment 1	1.15 (1.03)	−0.50 (1.11)	0.90 (1.11)	−0.40 (0 .99)
Experiment 2	2.34 (0.91)	1.41 (1.08)	2.27 (0.81)	1.70 (1.04)
Experiment 3	2.21 (0.84)	1.43 (1.05)	2.04 (0.87)	1.35 (0.95)

Standard deviations are in parentheses

#### Bias

Another way to assess response borrowing is to compute a signal-detection index of bias—*c* (hit rate plus false-alarm rate, both transformed into *z*-scores, divided by −2). This measure allows for evaluating participants’ propensity to respond ‘true’ across conditions, with a greater willingness to choose ‘true’ as a response reflected here in lower *c* values. Then, the difference in *c* scores between trials with a ‘true’ cue and those with a ‘false’ cue constitutes an index of response borrowing. Of interest here is whether this index of response borrowing differs across credible and noninformative sources.

The descriptive statistics for *c* are presented in Table 3. A 2 (cue type: true, false) × 2 (source: credible, noninformative) repeated-measures ANOVA performed on *c* scores revealed only a significant main effect of cue type, *F*(1, 71) = 66.77, *MSE* = 0.59, *p* < .001, ${\upeta}_{\textrm{p}}^2$= .485. This was caused by lower *c* scores—a greater willingness to respond ‘true’ to ‘true’-cued trials (*M* = −0.56, *SD* = 0.46) than to ‘false’-cued trials (*M* = 0.18, *SD* = 0.53). The main effect of source was not significant, *F*(1, 71) = 2.40, *MSE* = 0.09, *p* = .126, ${\upeta}_{\textrm{p}}^2$= .033, and neither was the interaction, *F*(1, 71) = 1.85, *MSE* = 0.30, *p* = .178, ${\upeta}_{\textrm{p}}^2$= .025. These results demonstrate that participants chose ‘true’ as their answer more often when provided with a ‘true’ than a ‘false’ cue, supporting the observation from the accuracy analysis that participants borrowed responses from external sources and presented them in lieu of their own responses. Crucially, as evidenced by the lack of a significant interaction, this difference in the propensity to respond ‘true’ did not depend on the particular source that provided the cue.

**Table 3:** Mean values of c across source conditions and cue type in Experiments 1–3

Experiment	Credible source		Noninformative source	
	‘true’	‘false’	‘true’	‘false’
Experiment 1	−0.63 (0.56)	0.20 (0.62)	−0.49 (0.59)	0.16 (0.56)
Experiment 2	−0.49 (0.36)	−0.04 (0.42)	−0.47 (0.38)	−0.19 (0.37)
Experiment 3	−0.58 (0.37)	−0.16 (0.41)	−0.56 (0.36)	−0.23 (0.35)

Standard deviations are in parentheses

#### Source identification

The numbers and percentages of participants’ answers to the postexperimental question are presented in Table 4. After the main test, participants decided which of the two sources was more reliable. An answer of one participant was not recorded because of an error in the experimental procedure. Forty-seven participants correctly indicated the credible source, and 24 participants chose the noninformative source. This difference was significant when assessed with a two-tailed sign test, *p* = .009.^4^

**Table 4:** Numbers and percentages of participants who chose the credible source, noninformative source, or neither source as more accurate in the postexperimental questionnaire in Experiments 1–3

Experiment	Credible		Noninformative		Neither	
	*N*	%	*N*	%	*N*	%
Experiment 1	47	66.2	24	33.8	–	–
Experiment 2	29	40.3	6	8.3	37	51.3
Experiment 3	28	38.9	11	15.3	33	45.8

Together, the results of Experiment 1 provide a clear demonstration of response borrowing in a task requiring truth assessments for trivia statements. However, this pattern of response borrowing was the same for both sources. This indiscriminate response borrowing occurred even though participants had a proper basis for inferring source credibility. The inclusion of easy questions in the first test offered the information necessary for assessing which of the sources provided cues of better quality, which was evidenced in the results of the postexperimental questionnaire. Forced to choose the more credible source, participants were often able to correctly identify the source providing more accurate cues. However, these explicit judgments of source credibility did not find their reflection in the indirect measure of response borrowing in the main experimental task. In this, the results of the present experiment closely follow the results of Jaeger et al. (2012), who used a word recognition task and also documented a similar dissociation across their measures of perceived source credibility and participants’ behavior as reflected by their response borrowing.

## Experiment 2

In Experiment 1, participants borrowed responses from the credible and noninformative source to the same extent, despite being able to explicitly identify the more credible source. One reason for this pattern of indiscriminate response borrowing could be that participants did not have a sufficiently strong internal standard for assessing credibility. Experiment 1 exposed participants to easy questions, for which a sufficiently strong internal reference point was available, but the number of such easy questions was limited. As a result, participants could develop some inkling as to the relative credibility of the sources, but they might have been insufficiently confident in their own assessments to allow them to govern response borrowing for a large number of difficult questions faced in the main experimental task. Only when asked directly about relative credibility could participants have used their less-than-confidently held opinions regarding the two sources to reveal accurate perceptions of source credibility. In Experiment 2, we attempted to create conditions more amenable to formulating strong opinions on source credibility. For this purpose, we removed the easy questions phase from the design and instead focused on providing participants a proper internal standard for judging the validity of external cues for difficult questions. We then presented participants at the beginning of the experiment with all questions together with their answers so that they could learn that information for a future test. We reasoned that if participants were able to learn and then retrieve the answers to at least some of these questions, they could use this newly gained knowledge as their internal standard and this should give them ample of opportunity to formulate strong opinions regarding the credibility of both sources in the course of the trivia task.

### Method

#### Participants

Seventy-two students (55 female) of various universities based in Kraków, Poland, between the ages of 18 and 38 years (*M* = 25.10, *SD* = 4.78) participated in the study. They received either monetary compensation or course credit for their participation.

#### Materials, design, and procedure

The set of 288 difficult questions from Experiment 1 was used in this experiment. The main difference between the present experiment and Experiment 1, apart from the exclusion of all easy questions from the procedure, lies in the addition of a study phase before the test. In this study phase, all 288 questions were presented in a random order together with their correct answers. There were two conditions of learning, implemented in two experimental groups with 36 participants each. In the reading group, questions and answers were displayed one-by-one on a computer screen for 15 s, with a 500-ms interstimulus interval (ISI). In the guessing group, participants were first shown a question for 12 s and asked to type in the answer within that time. They were encouraged to answer all questions, guessing if necessary. Then, participants were shown the question and the answer together for 3 s, before progressing to the next questions after a 500-ms ISI. This manipulation at study was implemented to vary the level of learning, following previous studies showing better memory after guessing than reading (e.g., Kornell, 2014). However, in the present study we failed to observe any differences across the two learning methods when we compared performance in the baseline, uncued condition. The learning condition also did not enter into any interactions with the cueing or source manipulations. Consequently, we collapsed our data across the learning method manipulation and we do not discuss it further. Thus, as in Experiment 1, all analyses reported below are based on within-participant comparisons.^5^

After the study phase, the procedure progressed to the test phase, which was the same as the second test in Experiment 1. Upon completion of the main task, participants were administered a postexperimental questionnaire in which they were asked to identify the more credible source. A change was introduced here compared with Experiment 1, as participants could now choose between three response options, identifying one of the sources or responding ‘neither’. The ‘neither’ option was added to filter out participants who either perceived both sources as equally credible or who did not have enough knowledge to differentiate between the sources. In this way, we hoped to limit the number of guesses to this final question, which could artificially inflate the numbers of correct identifiers.

### Results

#### Accuracy

We analyzed *d′* scores as a function of cuing condition (cued-credible, cued-noninformative, uncued) with a repeated-measures ANOVA (see Table 1 for the descriptive statistics). This revealed significant differences between conditions, *F*(2, 142) = 6.30, *MSE* = .15, *p* = .002, ${\upeta}_{\textrm{p}}^2$= .081. Performance was higher when cues were provided by the credible source compared with the uncued condition, *t*(71) = 3.34, *SE* = 0.06, *p* = .001, *d* = 0.23. However, there was no significant difference between the cued-noninformative and uncued conditions, *t*(71) = 0.04, *SE* = 0.07, *p* = .967, *d* = 0.01, a pattern of results which replicates the findings from Experiment 1.

Then, as in Experiment 1, we assessed whether *d′* increases after valid versus invalid cueing (see Table 2 for the descriptive statistics). A 2 (cue validity: valid, invalid) × 2 (source: credible, noninformative) repeated-measures ANOVA performed on *d′* scores indicated a significant effect of cue validity, *F*(1, 71) = 58.40, *MSE* = 0.69, *p* < .001, ${\upeta}_{\textrm{p}}^2$= .451. Participants’ test performance was higher after valid (*M* = 2.31, *SD* = 0.78) than after invalid cuing (*M* = 1.56, *SD* = 0.97). The main effect of source was not significant, *F*(1, 71) = 3.34, *MSE* = 0.24, *p* = .072, ${\upeta}_{\textrm{p}}^2$= .045, but the interaction was, *F*(1, 71) = 6.28, *MSE* = 0.39, *p* = .015, ${\upeta}_{\textrm{p}}^2$ = .081. Valid cues, in comparison to invalid ones, increased participants’ performance when presented both by the credible, *t*(71) = 7.12, *SE* = 0.13, *p* < .001, *d* = 0.93, and the noninformative source, *t*(71) = 5.00, *SE* = 0.11, *p* < .001, *d* = 0.60. However, this increase in performance after receiving valid cues was more substantial for the credible (Δ*d′* = 0.93) than for the noninformative source (Δ*d′* = 0.57).

#### Bias

A 2 (cue type: true, false) × 2 (source: credible, noninformative) ANOVA performed on *c* scores (see Table 3 for descriptive statistics) yielded a significant main effect of cue type, *F*(1, 71) = 53.44, *MSE* = 0.18, *p* < .001, ${\upeta}_{\textrm{p}}^2$= .429. As in Experiment 1, participants responded ‘true’ more often when the cue also said ‘true’ (*M* = −0.48, *SD* = 0.30) than when it said ‘false’ (*M* = −0.11, *SD* = 0.34), indicating response borrowing from external sources. The main effect of source was not significant, *F*(1, 71) = 3.52, *MSE* = 0.09, *p* = .065, ${\upeta}_{\textrm{p}}^2$= .047. There was, however, a significant interaction between cue type and source, *F*(1, 71) = 4.91, *MSE* = 0.10, *p* = .030, ${\upeta}_{\textrm{p}}^2$= .065. Even though participants borrowed responses from both sources, *t*(71) = 6.68, *SE* = 0.07, *p* < .001, *d* = 1.15, for the credible source, and *t*(71) = 5.00, *SE* = .06, *p* < .001, *d* = 0.76, for the noninformative source, the interaction arose because participants relied on the credible source to a greater extent (Δ*c* = 0.45) than on the noninformative one (Δ*c* = 0.28). This crucial difference between the results of the present experiment and Experiment 1 shows that the learning phase was successful in making participants attune their response borrowing to the actual credibility of the sources.

#### Source identification

The distribution of participants’ answers to the postexperimental question was not random, *χ*^2^(2) = 21.58, *p* < .001 (see Table 4 for the descriptive statistics). When we restricted our analysis to those who decided to pick one source over another, more participants (*n* = 29) correctly identified the credible source than wrongly opted for the noninformative source (*n* = 6), and this difference was significant when assessed with a two-tailed sign test, *p* < .001. Thus, the measure of explicit source credibility assessment shows that at least for those participants who indicated a difference in reliability between the two sources, the results of the postexperimental questionnaire were consistent with the response borrowing patterns.

Together, the results of Experiment 2 provide a clear demonstration of a crucial role of credibility inference for responding in a trivia task. This time not only were participants able to correctly name the credible source when explicitly asked to do so, but they were also able to attune their response borrowing depending on the actual source credibility. In other words, participants were able to limit—although only to some extent—their reliance on the source which provided responses of no informational value. These results stand in contrast both to the results of Experiment 1, where only explicit source credibility assessments (but not response borrowing) differentiated between credible and noninformative sources, and from previous studies using the same paradigm with a word recognition rather than a trivia task (Jaeger et al., 2012; Zawadzka et al., 2016). However, the present results are consistent with recent evidence from the domain of perception (Pescetelli & Yeung, 2021), which documented a similarly successful modulation of response borrowing depending on source credibility. Arguably, thus, it is not the immediate availability of correct responses in the environment that determines the use of credibility inferences in service of shaping one’s own responding. Rather, what seems to matter for credibility-informed response borrowing is the strength of internal evidence that affords a basis for systematic assessment of accuracy of multiple responses provided by external sources.^6^ When this strength of evidence is high, credibility monitoring by and large succeeds and allows for discriminate response borrowing.

## Experiment 3

In Experiment 3, the trivia test was postponed for 24 hours after the study phase. This modification is important from a practical and theoretical perspective. From the practical perspective, outside psychological laboratories, there is usually a time gap between learning new facts and being confronted with additional information. Therefore, the experiment was designed to check if participants would be able to infer source credibility and modulate response borrowing accordingly after a delay. From the theoretical perspective, we assumed that credibility inference in Experiment 2 was successful due to strong memory representations of correct answers, providing a good reference point for judging the accuracy of the cues provided by external sources. If so, then inserting a delay between learning and testing could be expected to reduce the strength of memory representations and thus impede participants’ ability to correctly infer source credibility. We would thus expect successful credibility inference to be at least reduced, or perhaps eliminated, in the present experiment, confirming the critical role of memory strength in this process.

### Method

#### Participants

Seventy-two students (55 female) of various high schools and universities based in Kraków, Poland, between the ages 18 and 31 years (*M* = 22.19, *SD* = 2.88), participated in this experiment. They received either monetary compensation or course credit for their participation.

#### Materials, design, and procedure

The same materials as in Experiment 2 were used. The design was the same as in Experiment 2, bar the addition of a 24-hour delay (discrepancies of ±2 hours were accepted) between the study and test phases of the experiment. The procedure was also the same as in Experiment 2, with the sole exception being that participants were required to return to the laboratory within an allotted time slot the next day to complete the final test. We also included a manipulation of learning—via reading or guessing with feedback—in the current procedure, yet it again failed to result in any differences in performance for the control, uncued condition, or any interactions with cuing or source manipulations, and it is thus not discussed further.

### Results

#### Accuracy

We analyzed *d′* scores as a function of cuing condition (cued-credible, cued-noninformative, uncued) using a one-way ANOVA (see Table 1 for the descriptive statistics). This ANOVA revealed significant differences between the conditions, *F*(2, 142) = 15.03, *MSE* = 0.17 , *p* < .001 , ${\eta}_{\textrm{p}}^2$ = .175. Overall, performance was higher when cues were provided by the credible source compared with the uncued condition, *t*(71) = 3.88, *SE* = 0.07, *p* < .001, *d* = 0.37, while there was no significant difference between the cued-noninformative and uncued conditions, *t*(71) = 1.18, *SE* = 0.07, *p* = .244, *d* = 0.10.

Then, we assessed whether *d′* increased after valid versus invalid cueing (see Table 2). This time, a 2 (cue validity: valid, invalid) × 2 (source: credible, noninformative) repeated-measures ANOVA performed on *d′* scores indicated only a significant main effect of cue validity, *F*(1,71) = 39.03, *MSE* = 0.98, *p* < .001, ${\upeta}_{\textrm{p}}^2$= .355. Participants’ test performance was higher after valid (*M* = 2.12, *SD* = 0.80) than after invalid cueing (*M* = 1.39, *SD* = 0.90). The main effect of source, *F*(1, 71) = 3.82, *MSE* = 0.30, *p* = .055, ${\eta}_{\textrm{p}}^2$= .051, was not significant and neither was the interaction, *F*(1, 71) = 0.60, *MSE* = 0.28, *p* = .442, ${\eta}_{\textrm{p}}^2$ = .008.

#### Bias

A 2 (cue type: true, false) × 2 (source: credible, noninformative) ANOVA performed on *c* scores yielded again a significant main effect of cue type, *F*(1, 71) = 50.24, *MSE* = 0.21, *p* < .001, ${\upeta}_{\textrm{p}}^2$ = .414, with lower *c* scores when items were presented with a ‘true’ (*M* = −0.57, *SD* = 0.31) rather than a ‘false’ cue (*M* = −0.19, *SD* = 0.34), which indicates response borrowing from external sources (see Table 3). Contrary to Experiment 2, this time neither the main effect of source, *F*(1, 71) = 0.98, *MSE* = 0.07, *p* = .325, ${\eta}_{\textrm{p}}^2$ = .014, nor the interaction of cue and source, *F*(1, 71) = 1.89, *MSE* = 0.07, *p* = .173, ${\eta}_{\textrm{p}}^2$ = .026, was significant. The nonsignificant interaction here indicates that the degree of borrowing was comparable when cued by the credible and the noninformative source, mirroring the results obtained in Experiment 1.

#### Source identification

The distribution of source identification answers was not random, χ^2^(2) = 11.08, *p* = .004 (see Table 4). As in Experiment 2, upon exclusion from the analysis the participants who picked the ‘neither’ option, it turned out that more participants (*n* = 28) correctly identified the credible source than incorrectly marked the noninformative source as credible (*n* = 11), *p* = .01 when assessed with a two-tailed sign test. Thus, even after a delay, a greater number of participants was able to explicitly identify the credible source than misidentify the noninformative source as providing more accurate information.

The results of Experiment 3 remain close to those of Experiment 1, but differ in crucial aspects from the results of Experiment 2. The introduction of a delay between learning trivia and assessing the truth value of these trivia statements at test resulted again in indiscriminate response borrowing from credible and noninformative sources, while still allowing more participants to explicitly identify the more credible source in the postexperimental questionnaire than the noninformative source. These results again suggest the possibility of a dissociation across explicit judgments of reliability and an indirect measure of actual reliance on information provided by external sources differing in credibility. While Experiment 2 showed that such a dissociation is not inevitable, the present experiment demonstrates that very specific conditions are necessary for the measure of response borrowing to reveal the effect of source credibility. This again suggests that while participants can sometimes successfully infer the relative credibility of the sources, they may not be confident enough in their inferences as to allow them to guide response borrowing. We suggest that conditions that are necessary for such high-confidence inferences include a very strong internal memory standard for judging the accuracy of external sources’ responses—present when participants were freshly exposed to correct answers just before the test in Experiment 2, but already fading when the testing phase was delayed in Experiment 3. However, it should also be noted that the conclusions regarding the role of delay rest here only on comparisons across different experiments so they need to be treated as tentative until high-powered follow-up experiments are conducted to confirm them.

## General discussion

The present study assessed people’s ability to, first, infer the credibility of external sources and, second, use their inferences regarding source credibility to attune their reliance on responses provided by these sources when judging the validity of trivia statements. Are people able to use their own knowledge to discover that one source provides them with more credible information than another? The results of three experiments show the answer to this question to be ‘yes’. Whether judgments of source credibility needed to be based on a relatively small sample of easy trivia questions to which responses were generally known (Experiment 1), or on a large sample of difficult questions for which correct answers were either learned just before (Experiment 2), or a day before the test (Experiment 3), there was always at least some indication that participants were able to explicitly identify the more credible source. However, such correct identifications were far from universal. When in Experiment 1 participants were forced to provide their relative credibility judgments, 66% of them were correct. This number could have been, however, inflated by those who picked the credible source merely by guessing. When in Experiments 2 and 3 participants’ responses were not forced and the option ‘neither’ was available, only 40% and 39%, respectively, provided correct identifications. Thus, although deriving information regarding source credibility based solely on the responses provided by this source and one’s own internal standard for assessing accuracy of these responses is clearly possible (see also Collins et al., 2018), it is not an easy task even when one is confronted with a vast number of responses from these sources.

Are people also able to attune their response borrowing and rely on sources that provide them with useful information as opposed to random responses? The present results suggest that only to some extent and under very restricted conditions. First, all experiments clearly showed response borrowing not only from a source that provided by and large accurate responses but also from one that responded randomly. This occurred even in Experiment 1, when this noninformative source was initially seen to respond at random to a set of easy questions, for which participants’ accuracy was about 90%. Despite being confronted with a number of responses from the noninformative source that must have clearly contradicted what participants already knew, the perceived credibility of this source was not undermined to the extent that would prevent participants from borrowing other responses from it. These results resemble closely those of Jaeger et al. (2012), who also showed response borrowing for sources providing cues of random accuracy, and remain consistent with other research suggesting that borrowing responses from external sources is a largely automatic process that is to some extent beyond participants’ control (Selmeczy & Dobbins, 2017).

Second, despite some ability to identify the more credible source revealed in explicit judgments collected after the main experimental task, during the task itself participants in Experiments 1 and 3 borrowed responses from both sources at a similar rate. Only in Experiment 2, where participants learned correct answers for all questions just before these questions were presented again with cues from external sources, were participants able to differentiate their response borrowing across sources differing in credibility, borrowing responses somewhat less often from the noninformative rather than the credible source. It seems, thus, that differentiated response borrowing is much more difficult to achieve than a simple identification of the more credible source.

The present study built on recent considerations of the role of source credibility in various situations involving social influence. Recent theories of credibility stress that there is an intricate relationship between the analysis of the content of the message and perception of how credible the source of this message is (Collins et al., 2018). Here, we described this relationship as bidirectional, in that the perceived credibility of a source can affect how the content of the message is processed, but the analysis of the content equally affects the perceived credibility of its source. This relationship is also dynamic, in that credibility inferred on the basis of content analysis of the messages from a particular source affects processing of the further messages this source delivers. The results of the present study provide strong evidence for a bidirectional nature of the message-source relationship as they demonstrate that participants can formulate their explicit judgments of source credibility based solely on the responses of these sources in a trivia task. In this they confirm previous studies demonstrating how people use their own internal standards of either perception (Pescetelli & Yeung, 2021) or memory (Jaeger et al., 2012) to infer credibility of external sources. As for the dynamic nature of this relationship, the results of Experiment 2 provide evidence that a process in which content analysis informs credibility assessments, which in turn affect content analysis, is also possible, but then the results of Experiment 1 and 3 underscore that such a dynamic relationship may easily break down.

The outstanding question remains of why differentiated response borrowing is so difficult to achieve. By using the memory task we initially ventured to assess whether the requirement to retrieve answers from memory interferes with remembering the tally of agreements and disagreements between external messages and one’s internal standard. By documenting differentiated response borrowing in Experiment 2, we demonstrated, however, that even if this interference is present, it does not fatally impede credibility-informed information processing. What our results suggest instead is that a crucial factor for emergence of differentiated response borrowing is the strength of the internal evidence required for inferring source credibility. While this evidence was strong in Experiment 2, where due to a recent study experience participants could know answers to almost all questions for which the two sources provided their own responses, it could have been considerably weaker in both Experiment 1, where participants could use their own knowledge to assess source accuracy only for a small minority of easy questions, and in Experiment 3, where answers to all questions were learned, but could have been easily forgotten in a 24-hour delay across learning and testing sessions. This crucial role of the internal standard may explain why a previous study assessing response borrowing in the domain of perception, where the internal standard is built on the basis of perceiving patterns in the immediate environment, showed clear evidence for response borrowing attuned to actual source credibility (Pescetelli & Yeung, 2021), while a similar study using a word recognition paradigm—with participants having relatively poor memory for the learned materials—showed no such differentiated response borrowing (Jaeger et al., 2012).^7^

Given, however, participants’ preserved ability to infer source credibility—as evidenced by explicit source identifications—it seems that the strength of the internal standard is not the only factor that determines whether differentiated response borrowing is likely to emerge. Why did participants in Experiments 1 and 3 fail to attune their response borrowing to the credibility of the source even though in principle they had at least some inkling as to which source was more credible? One related observation from the present experiments could be that borrowing responses from the credible source boosts one’s performance, while borrowing them from the source providing random responses does not lead to any costs to performance. This pattern has been previously assigned to the workings of the low-confidence outsourcing mechanism (Jaeger et al., 2012; see also Koop et al., 2021), where responses are borrowed only when the accuracy of participants’ own responses is judged by them to be at chance. When participants instead of randomly guessing at the correct response decide to fall back on a response provided by an external source which turns out also to be a random guess, then one random response is substituted for another random response. The low-confidence outsourcing mechanism may be important for the pattern of indiscriminate response borrowing because it essentially takes away the cost of relying on a noninformative source. Participants may rightly believe that even though a given source is not that credible, there still may be some informational value associated with its responses that makes it worthwhile to borrow them from time to time. This argument does not preclude the pattern of differentiated response borrowing observed in Experiment 2, where participants could increase their reliance on an external source which they strongly felt to be credible also in those cases in which they judged their own response to be at least somewhat likely to be correct (see also Horry et al., 2012).

The aforementioned considerations lead to a prediction that differentiated response borrowing would emerge when participants judge a particular source not only as relatively less credible but instead as providing responses that truly cannot be trusted to be accurate at all. This is indeed a pattern that was previously observed by Jaeger et al. (2012, Experiment 2) with a word recognition task, when a noninformative source provided responses that were accurate only 25% of the time. It should be noted here, however, that such a manipulation of source credibility fundamentally changes the task participants face. In the present study, we varied accuracy between 83% for the credible source and 50% for the noninformative source. This can be described as a manipulation of expertise: external sources were either credible ‘experts’ in assessing the validity of trivia statements or had no relevant knowledge for the assessment of such statements. Arguably, using a source with accuracy below 50% changes the manipulation to one of trustworthiness: The source has knowledge regarding trivia but uses it to present lies rather than accurate information. Expertise and trustworthiness are often considered two separate aspects of credibility (Guillory & Geraci, 2013; Lombardi et al., 2014), and from this perspective, our study demonstrates the possibility and limitations of differentiated response borrowing when facing sources of various levels of expertise. Follow-up studies would have to extend our findings to the domain of trustworthiness.

## Appendix 1

At the end of each experiment, participants chose which source they assumed was more accurate. It was of interest whether correct identifiers (those who picked the credible source in the final question) would exhibit a different pattern of response borrowing than incorrect identifiers (those who picked the noninformative source or chose the ‘neither’ option). Thus, we checked whether correct identifiers borrowed responses in the main test from the source which provided cues of no informative value. If so, the follow up-question was whether only correct identifiers would exhibit differentiated patterns of response borrowing (i.e., reliance to a greater extent on the responses provided by the credible source than the noninformative one). We examined response borrowing using signal-detection measures of discrimination *d′* and bias *c* (see Appendix Tables 5 and 6 for the descriptive statistics).

### Experiment 1

Two 2 (cue type: true, false) × 2 (source: credible, noninformative) repeated-measures ANOVAs on *c′* scores revealed significant main effects of cue—*F*(1, 46) = 48.59, *MSE* = 0.64, *p* < .001, ${\upeta}_{\textrm{p}}^2$ = .514, and *F*(1, 23) = 16.89, *MSE* = 0.42, *p* < .001, ${\upeta}_{\textrm{p}}^2$= .423, for correct and incorrect identifiers, respectively. This pattern indicates response borrowing from external sources in both groups. The main effects of source was not significant for either correct, *F*(1, 46) = 3.63, *MSE* = 0.07, *p* = .063, ${\upeta}_{\textrm{p}}^2$ = .073, and incorrect identifiers, *F*(1, 23) = 0.01, *MSE* = 0.12, *p* = .927, ${\upeta}_{\textrm{p}}^2$ < .001. A significant interaction of cue type and source was obtained only for correct identifiers, *F*(1, 46) = 4.77, *MSE* = 0.26, *p* = .034, ${\upeta}_{\textrm{p}}^2$ = .094, but not for incorrect identifiers: *F*(1, 23) = 0.20, *MSE* = 0.36, *p* = .663, ${\upeta}_{\textrm{p}}^2$ = .008. Correct identifiers relied more on the responses provided by the credible source—Δ*c* = 0.97, *t*(46) = 7.00, *SE* = 0.14, *p* < .001, *d* = 1.66—than the noninformative one (Δ*c* = 0.65, *t*(46) = 4.73, *SE* = 0.14, *p* < .001, *d* = 1.15).

An analysis of response borrowing using *d′* scores mimicked these observations. Two 2 (cue validity: valid, invalid) × 2 (source: credible, noninformative) repeated-measures ANOVAs, separately for correct and incorrect identifiers, showed main effects of cue validity: *F*(1, 46) = 48.72, *MSE* = 2.53, *p* < .001, ${\upeta}_{\textrm{p}}^2$ = .514, and *F*(1, 23) = 16.89, *MSE* = 1.68, *p* < .001, ${\upeta}_{\textrm{p}}^2$ = .423, respectively. Thus, test performance was higher after valid than after invalid cuing indicating that participants followed cues from the external sources. There was no significant main effect of source for either correct, *F*(1, 46) = 2.82, *MSE* = 0.26, *p* = .100, ${\upeta}_{\textrm{p}}^2$ = .058, and incorrect identifiers, *F*(1, 23) = 0.21, *MSE* = 0.32, *p* = .651, ${\upeta}_{\textrm{p}}^2$ = .009. However, a significant interaction of cue validity and source, *F*(1, 46) = 4.68, *MSE* = 1.04, *p* = .036, ${\upeta}_{\textrm{p}}^2$ = .092, revealed that only for correct identifiers were the differences in *d′* scores between valid and invalid cueing conditions higher for the credible source—Δ*d′* = 1.94, *t*(46) = 7.01, *SE* = 0.28, *p* < .001, *d* = 1.76—than the noninformative source—Δ*d′* = 1.30, *t*(46) = 4.73, *SE* = 0.27, *p* < .001, *d* = 1.23. For incorrect identifiers, the same interaction was not significant, *F*(1, 23) = 0.20, *MSE* = 1.43, *p* = .663, ${\upeta}_{\textrm{p}}^2$ = .008.

Thus, correct identifiers borrowed more responses from the credible than noninformative source, yet for incorrect identifiers there was no such difference. Importantly, however, even for correct identifiers response borrowing from the noninformative source was not eliminated.

### Experiment 2

In Experiment 2, 29 participants correctly identified the credible source and 43 wrongly opted for the noninformative source or chose the ‘neither’ option in the final question. We examined the borrowing pattern separately for these groups. Two 2 (cue type: true, false) × 2 (source: credible, noninformative) repeated-measures ANOVAs on *c* scores revealed significant main effects of cue type—*F*(1, 28) = 27.47, *MSE* = 0.19 , *p* < .001, ${\upeta}_{\textrm{p}}^2$ = .495, and *F*(1, 42) = 26.32, *MSE* = 0.17, *p* < .001, ${\upeta}_{\textrm{p}}^2$= .385—for correct and incorrect identifiers, respectively. This pattern indicates response borrowing from external sources in both groups. The main effect of source was not significant in any of the analyses: *F*(1, 28) = 1.58, *MSE* = 0.07, *p* = .219, ${\upeta}_{\textrm{p}}^2$ = .053—for correct identifiers, and *F*(1, 42) = 1.93, *MSE* = 0.10, *p* = .172, ${\upeta}_{\textrm{p}}^2$ = .044—for incorrect ones. Importantly, a significant interaction of cue type and source was obtained only for correct identifiers, *F*(1, 28) = 11.00, *MSE* = 0.11, *p* = .003, ${\upeta}_{\textrm{p}}^2$ = .282; incorrect identifiers: *F*(1, 42) = 0.01, *MSE* = 0.70, *p* = .933, ${\upeta}_{\textrm{p}}^2$ < .001. Thus, only correct identifiers manifested some degree of source discrimination and relied more on the responses provided by the credible source—Δ*c* = .63, *t*(28) = 5.66, *SE* = 0.11, *p* < .001, *d* = 1.59—than the noninformative one—Δ*c* = .22, *t*(28) = 2.39, *SE* = 0.09, *p* = .024, *d* = 0.60.

As in Experiment 1, the analysis of response borrowing using *d′* scores after valid versus invalid cueing indicated the same pattern as in the case of *c* scores. Two 2 (cue validity: valid, invalid) × 2 (source: credible, noninformative) repeated-measures ANOVAs revealed significant main effects of cue validity: *F*(1, 28) = 27.65, *MSE* = 0.77, *p* < .001, ${\upeta}_{\textrm{p}}^2$ = .497, for correct identifiers, and *F*(1, 42) = 30.59, *MSE* = 0.65, *p* < .001, ${\upeta}_{\textrm{p}}^2$ = .421, for incorrect identifiers. The main effect of source was not significant: *F*(1, 28) = 0.54, *MSE* = 0.19, *p* = .469, ${\upeta}_{\textrm{p}}^2$ = .019, for correct identifiers, and *F*(1, 42) = 2.89, *MSE* = 0.28, *p* = .097, ${\upeta}_{\textrm{p}}^2$ = .064, for incorrect identifiers. Crucially, only for correct identifiers was the difference in *d′* scores after valid versus invalid cueing higher for the credible source—Δ*d′* = 1.27, *t*(28) = 5.68, *SE* = 0.22, *p* < .001, *d* = 1.25—than for the noninformative source—Δ*d′* = 0.44, *t*(28) = 2.39, *SE* = 0.18, *p* = .024, *d* = 0.47—as confirmed by a significant interaction of identification accuracy and cue validity, *F*(1, 28) = 11.02, *MSE* = 0.45, *p* = .003, ${\upeta}_{\textrm{p}}^2$ = .282. For incorrect identifiers, this interaction was not significant, *F*(1, 42) = 0.12, *MSE* = 0.29, *p* = .735, ${\upeta}_{\textrm{p}}^2$ = .003.

The pattern of discriminate borrowing found in the overall analysis in Experiment 2 can be mainly attributed to the fact that correct identifiers were able to determine correctly which source was more accurate and relied more on cues provided by the credible source that by the noninformative one. However, even those participants still borrowed responses from the noninformative source.

### Experiment 3

We assessed response borrowing separately for correct (*n* = 28) and incorrect (*n* = 44) identifiers in the same way as in Experiment 2. Two 2 (cue type: true, false) × 2 (source: credible, noninformative) repeated-measures ANOVAs on *c* scores revealed significant main effects of cue type, *F*(1, 27) = 48.76 , *MSE* = 0.20, *p* < .001, ${\upeta}_{\textrm{p}}^2$ = .644, and *F*(1, 43) = 15.80, *MSE* = 0.16, *p* < .001, ${\upeta}_{\textrm{p}}^2$ = .269, for correct and incorrect identifiers, respectively. These results indicate that participants borrowed responses from external sources. The main effects of source were not significant for both correct, *F*(1, 27) = 0.48, *MSE* = 0.08, *p* = .493, ${\upeta}_{\textrm{p}}^2$ = .018, and incorrect identifiers, *F*(1, 43) = 0.49, *MSE* = 0.06, *p* = .489, ${\upeta}_{\textrm{p}}^2$ = .011. Again, there was a significant interaction of cue type and source only for correct identifiers, *F*(1, 27) = 6.19, *MSE* = 0.11, *p* = .019, ${\upeta}_{\textrm{p}}^2$ = .186, but not for incorrect identifiers, *F*(1, 43) = 0.92, *MSE* = 0.04, *p* = .343, ${\upeta}_{\textrm{p}}^2$ = .021. This shows that, as in Experiment 2, correct identifiers manifested the pattern of discriminate response borrowing, with Δ*c* equal to .75 for the credible source, *t*(27) = 6.54, *SE* = 0.11, *p* < .001, *d* = 1.72, and .44 for the noninformative source, *t*(27) = 4.63, *SE* = 0.10, *p* < .001, *d* = 1.18. Thus, in Experiment 3, we replicated observations from Experiment 1 and 2 regarding the different pattern of responses borrowing in the case of correct versus incorrect identifiers.

For *d′* scores, two 2 (cue validity: valid, invalid) × 2 (source: credible, noninformative) repeated-measures ANOVAs indicated only main effects of cue validity: *F*(1, 27) = 27.24, *MSE* = 1.13, *p* < .001, ${\upeta}_{\textrm{p}}^2$ = .502, for correct identifiers and, *F*(1, 43) = 15.19, *MSE* = 0.80, *p* < .001, ${\upeta}_{\textrm{p}}^2$ = .261, for incorrect identifiers. The main effects of source were not significant, *F*(1, 27) = 1.67, *MSE* = 0.32, *p* = .207, ${\upeta}_{\textrm{p}}^2$ = .058, for correct identifiers and *F*(1, 43) = 2.10, *MSE* = 0.30, *p* = .154, ${\upeta}_{\textrm{p}}^2$ = .047 for incorrect identifiers. Importantly, neither for correct identifiers, *F*(1, 27) = 1.50, *MSE* = 0.46, *p* = .232, ${\upeta}_{\textrm{p}}^2$ = .053, nor for incorrect identifiers, *F*(1, 43) = 0.13, *MSE* = 0.16, *p* = .726, ${\upeta}_{\textrm{p}}^2$ = .003, were the interactions of cue validity and source significant, which means that the pattern of discriminate response borrowing in correct identifiers was not replicated.

**Table 5:** Mean values of c across source identification accuracy, source conditions, and cue types in Experiments 1–3

	Correct identifiers				Incorrect identifiers			
Experiment	Credible source		Noninformative source		Credible source		Noninformative source	
	‘true’	‘false’	‘true’	‘false’	‘true’	‘false’	‘true’	‘false’
Experiment 1	−0.69 (0.55)	0.29 (0.62)	−0.45 (0.55)	0.20 (0.58)	−0.47 (0.53)	0.02 (0.60)	−0.51 (0.63)	0.08 (0.53)
Experiment 2	−0.63 (0.38)	0.01 (0.42)	−0.48 (0.39)	−0.26 (0.35)	−0.39 (0.31)	−0.07 (0.42)	−0.46 (0.38)	−0.13 (0.38)
Experiment 3	−0.70 (0.39)	0.05 (0.48)	−0.58 (0.37)	−0.14 (0.38)	−0.50 (0.34)	−0.29 (0.29)	−0.55 (0.36)	−0.29 (0.32)

Standard deviations are in parentheses

**Table 6:** Mean values of d′ across source identification accuracy, source conditions, and cuing validity in Experiments 1–3

	Correct identifiers				Incorrect identifiers			
Experiment	Credible source		Noninformative source		Credible source		Noninformative source	
	Valid cues	Invalid cues	Valid cues	Invalid cues	Valid cues	Invalid cues	Valid cues	Invalid cues
Experiment 1	1.36 (1.06)	−0.59 (1.15)	0.91 (1.13)	−0.39 (0.98)	0.69 (0.80)	−0.29 (1.02)	0.85 (1.11)	−0.35 (0.97)
Experiment 2	2.72 (0.79)	1.45 (1.18)	2.36 (0.76)	1.92 (1.05)	2.09 (0.90)	1.38 (1.03)	2.20 (0.85)	1.55 (1.01)
Experiment 3	2.41 (0.97)	1.20 (1.05)	2.11 (0.95)	1.22 (0.93)	2.08 (0.72)	1.58 (1.03)	1.98 (0.82)	1.44 (0.96)

Standard deviations are in parentheses

## Appendix 2

In the guessing groups of Experiments 2 and 3, on average, participants provided 6.28% (*SD* = 3.61%) and 7.60% (*SD* = 4.56%) of correct answers in the study phases. Tables 7 and 8 present *d′* and *c* scores, respectively, for the two learning groups (guessing and reading) in Experiments 2 and 3, and Table 9 presents data on source identification choices for these groups.

**Table 7:** Mean values of d′ across cuing and learning conditions in Experiments 2 and 3

Experiment	Reliable source	Unreliable source	No source
Experiment 2			
Guessing	2.06 (0.85)	1.89 (0.73)	1.81 (0.83)
Reading	2.27 (0.83)	2.04 (0.99)	2.11 (0.91)
Experiment 3			
Guessing	2.05 (0.60)	1.54 (0.64)	1.68 (0.58)
Reading	1.97 (0.90)	1.77 (0.95)	1.79 (0.86)

Standard deviations are in parentheses

**Table 8:** Mean values of c across source and learning conditions and cue type in Experiments 2 and 3

Experiment	Reliable source		Unreliable source	
	‘true’	‘false’	‘true’	‘false’
Experiment 2				
Guessing	−0.46 (0.34)	−0.10 (0.40)	−0.46 (0.35)	−0.24 (0.34)
Reading	−0.51 (0.37)	0.02 (0.44)	−0.48 (0.41)	−0.14 (0.39)
Experiment 3				
Guessing	−0.62 (0.36)	−0.12 (0.43)	−0.62 (0.38)	−0.22 (0.38)
Reading	−0.54 (0.38)	−0.19 (0.39)	−0.51 (0.34)	−0.24 (0.32)

Standard deviations are in parentheses

**Table 9:** Numbers and percentages of participants who chose the reliable source, unreliable source, or neither source as more accurate in the postexperimental questionnaire as a function of learning condition in Experiments 2 and 3

Experiment	Reliable		Unreliable		Neither	
	*N*	%	*N*	%	*N*	%
Experiment 2						
Guessing	14	38.9	4	11.1	18	50.0
Reading	15	41.7	2	5.6	19	52.8
Experiment 3						
Guessing	16	44.4	2	5.6	18	50.0
Reading	12	33.3	9	25.0	15	41.7

## Footnotes

1 The effect of using messages from external sources as one own’s responses is often referred to in the literature as *conformity*. However, the term *conformity* has negative connotations, which are not always justified, given that presenting responses coming from a credible source as one’s own is likely to improve one’s performance. We thus here refer to the same effect in a more neutral way as *response borrowing*.

2 The percentages were the same as in our previous study (Zawadzka et al., 2016). The value of 50% constitutes chance-level performance. Responses from a source performing at chance should be of no informative value from the perspective of a participant, as participants’ uninformed guesses should also have accuracy at around 50%. The value of 83.3% was chosen because it reflected high but not perfect accuracy.

3 We thank an anonymous reviewer for suggesting these analyses.

4 We also analyzed the pattern of response borrowing separately for correct and incorrect identifiers. The results are presented in Appendix 1.

5 Interested readers will find in Appendix 2 additional descriptive statistics—including *d′* and *c* scores, as well as source identification choices made upon completion of the experimental procedure—presented separately for the guessing and reading groups of Experiments 2 and 3. We also report there the percentages of correct guesses provided in the study phase by participants in the guessing groups.

6 It is worth noting here the improvement of *d′* scores in the uncued condition from Experiment 1 (without a study phase) to Experiment 2 (which included a study phase): *d′* in those experiments equalled 0.25 and 1.96, respectively. This shows that the addition of the study phase greatly improved participants’ ability to answer the questions correctly, strengthening their internal evidence and facilitating its use it as a standard for assessing cue accuracy.

7 In order to further investigate whether the strength of the internal memory standard is linked to the ability to correctly identify the credible source, we conducted a series of *t* tests comparing performance on uncued trials for correct and incorrect identifiers in the postexperimental questionnaire. Interestingly, in none of the experiments did the difference in performance between the two groups reach statistical significance (lowest *p* = .11 in Experiment 2).
